# Nanoimprint Mold Consisting of an Adhesive Lap Joint between a Nanopatterned Metal Sleeve and a Carbon Composite Roll

**DOI:** 10.3390/nano13101685

**Published:** 2023-05-20

**Authors:** Amin Khaliq, Muhammad Ahmad Kamran, Myung Yung Jeong

**Affiliations:** Department of Cogno-Mechatronics Engineering, Pusan National University, Busan 46241, Republic of Korea; aaminkhaliq@pusan.ac.kr (A.K.); malik@pusan.ac.kr (M.A.K.)

**Keywords:** roll-to-roll nanoimprinting process, nano-patterns, adhesive joining, composite material, solar cells

## Abstract

Roll-to-roll nanoimprinting is an emerging technology that has revolutionized the sectors of flexible electronics, thin films, and solar cells with its high throughput. However, there is still room for improvement. In this study, a FEM analysis (in ANSYS) was carried out on a large-area roll-to-roll nanoimprint system in which the master roller consists of a large nanopatterned nickel mold joined to a carbon fiber reinforced polymer (CFRP) base roller using epoxy adhesive. Deflections and pressure uniformity of the nano-mold assembly were analyzed under loadings of different magnitudes in a roll-to-roll nanoimprinting setup. Optimization of deflections was performed with applied loadings, and the lowest deflection value was 97.69 nm. The adhesive bond viability was assessed under a range of applied forces. Finally, potential strategies to reduce deflections were also discussed, which can be helpful in increasing pressure uniformity.

## 1. Introduction

Adhesive joining is the process by which two adherends are joined together (same or different) via the application of a substance with adhesive properties to the joining surfaces of the two adherends. Engineers and inventors are trying innovative approaches to make engineering systems more efficient. One such method is to combine composite materials with metals via adhesive joining in different applications in order to reduce the weight without compromising on the mechanical integrity. This has resulted in increased efficiency in several systems, e.g., automobiles, aerospace, mobiles, microelectronics, etc. [1,2,3,4,5]. Glass-reinforced plastic (GRP) and carbon-fiber-reinforced plastic/polymer (CFRP) are the most prominent examples. In recent times, adhesive joining has been increasingly adopted in many industrial sectors and high-end applications, such as the automotive industry, aviation industry, defense sector industries, textile industry, mobile industry, footwear industry, the aerospace sector, and even in the medical sector owing to their privilege of joining two dissimilar substrates [6,7,8]. Details regarding different types of adhesive joint configuration and their pros and cons can be found in the articles [9,10].

Many studies have been performed by researchers on the effects of different types of loading on adhesively bonded cylindrical joint assemblies, which have been briefly mentioned here. Murakami and Sekiguchi analyzed cylindrical adhesive butt joint specimens with two epoxy adhesives (one brittle and one ductile), testing quasi-static combined tension and shear stress. They found that failure strains were dependent on strain rate in the case of brittle adhesives [11]. Ozenc and Sekercioglu investigated the effect of surface roughness on impact stress for adhesively bonded cylindrical components and found an optimum range of R-a values ranging from 1.5 to 2.5 mu m, with steel showing the highest impact stress while aluminum specimens showed the lowest [12]. Nemes and Lachaud improved the stress distribution model of adhesively joined cylindrical assemblies by including the radial stresses, due to which the model is able to better predict intensity and stress distribution [13]. Jean and Laurent performed 2D simulations on various geometries of cylindrical adhesive joints under tension and compression, which demonstrated that geometries could influence the stress concentration in the joints [14]. Xu and Li performed a 3D analysis of composite tubular adhesive joints by evaluating various adhesive and composite parameters. Their results were validated by the literature [15]. Kim and Chun simulated shear directional impact on adhesive tubular joints and found that the FE model showed good agreement with the experimental results [16]. Parashar et al. wrote in great detail about the different ways of adhesively joining FRP pipes, along with failure mechanisms under various loading conditions [17]. Hamoodi-Tabar et al. studied the long-term effects of temperature on shear stress distribution on adhesively joined tubular joints and showed that the value of shear stress is at maximum at lower temperatures [18].

**Figure 1:**
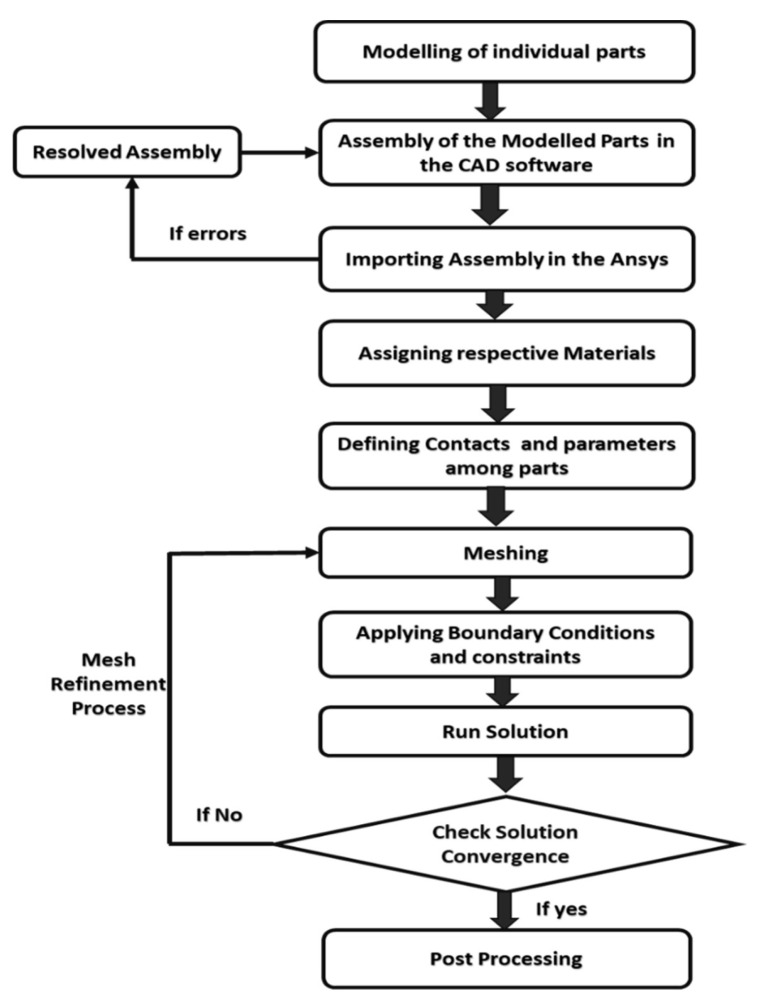
Hierarchy of instruction for the proposed process.

As well as experimental analyses, researchers studying adhesively bonded systems also use simulation tools. These commercial software programs have been helpful for predicting, assessing, and optimizing the behavior of the adhesively bonded system under observation. A brief description of such studies follows. Kim et al. performed shear directional impact analysis using the commercial software LS-DYNA to model the cohesive zone, and obtained results that were close to experimental results [16]. Hamoodi-Tabar et al. also validated the results of an experimental study on the long-term behavior of shear stress distribution with regard to temperature on adhesively bonded tubular joints using the Ansys simulation software [18]. Hou et al. also used LS-DYNA to model the failure characteristics of a single hat-shaped thin-walled tubular T-joint under impact loading with regard to different velocities [19]. Mustapha et al. used explicit simulation with ABAQUS to assess the mechanical strength and crushing behavior of the cylindrical shell fuselage under the impact of axial load and axial compression [20]. Bharti et al. also used Ansys as a simulation tool in order to optimize different joint parameters of adhesively joined tubular K-joints using failure criteria and FRP-composite ply stacking techniques. The results were then ratified by the available experimental records and FEM model results [21]. Baishya et al. studied the effect of different magnitudes of torsional loads on internally pressurized adhesively bonded tubular single lap joints using Ansys 14.0. The Tsai–Wu coupled stress criterion was used in order to assess the failures (adhesive or cohesive) of the TSLJ [22]. Kumaraswamidhas et al. carried out 3D stress analysis on tubular joints joined by epoxy adhesive using a triangular fillet model made up of laminated fiber-reinforced polymer under axial compressive stress. Ansys 18.1 was used to perform the analysis, and available FEM models were used for validation [23]. The basic steps involved in the simulation process are presented in Figure 1 in the form of a flowchart.

Since its inception in 1995, nanoimprint lithography has been a better alternative to other lithographic techniques due to its simple process, cost-effective nature, and high production rates [24]. Developments of miniaturized semiconductor devices to the nanoscale, oleophobic and hydrophobic surfaces, optical and photonic devices, along with nanomedicines and antimicrobial surfaces, are some of the important mentions which are achieved as fruits of advances in nanoimprinting [25,26,27,28,29,30,31,32,33,34].

**Figure 2:**
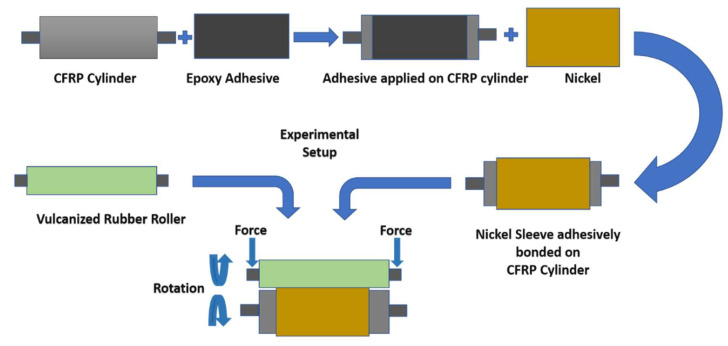
Schematic diagram of the roller fabrication.

Roll-to-roll (R2R) nanoimprint lithography has been dubbed the best nanoimprinting technique due to its ease of use, low cost, and high yield. Large area nanopatterning has great potential to significantly enhance the performance of many devices and create innovative and efficient products, such as solar cells, hydrophobic and oleophobic surfaces, large displays, etc. Although there are limitations to the development of large-area R2R nanoimprinting due to seam-related problems and deflection in the master mold assembly, researchers have been working to solve this problem [35]. Moreover, printing nanopatterns on a large area has been a challenge even without any seams present. The main reason for this is deflection/deformations in the master roller assembly. Whenever a large area nanopatterned mold is used in an R2R setup, deflections are present to the extent that seriously affects the product quality. These deflections are also the cause of pressure non-uniformity. In this study, a feasibility study for R2R nanoimprinting over a much larger area was performed in which a nickel nanopatterned mold was adhesively bonded to a carbon-fiber-reinforced polymer CFRP roll. Due to the large dimensions, bending in the master roller assembly cannot be avoided. In this study, to assess the large-scale roll-to-roll nanoimprint lithography setup behavior and related challenges, optimum magnitudes of loading were applied, and the deflection/bending was computed (in nanometers). Stresses in the adhesive layer between the mold and the CFRP roller during R2R operation were also evaluated, and the failure limits of the adhesive were also estimated. The schematic of the steps and experimental scenario is shown in Figure 2.

## 2. Materials and Methods

### 2.1. System Description

**Figure 3:**
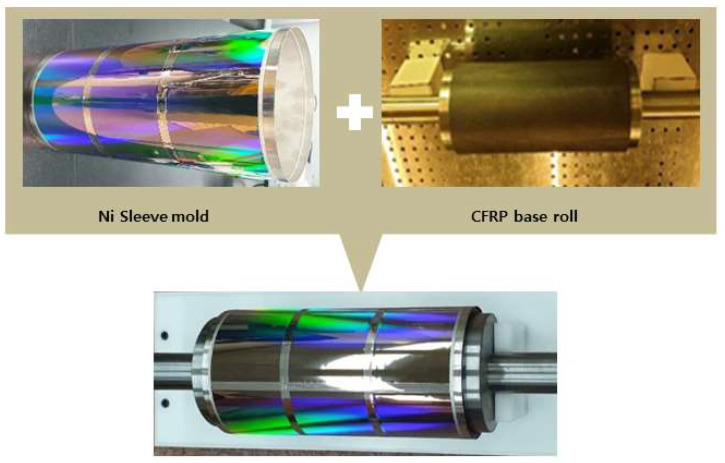
Nickel sleeve and CFRP base roll assembly.

In this study, a cylindrical adhesive lap joint between nickel and a carbon composite material (Epoxy Carbon UD (230 GPa) Prepreg) was analyzed under the application of nip loading. Properties of the materials, including nickel and Epoxy Carbon UD (230 GPa) Prepreg, were taken from the ANSYS material inventory. The fiber orientation of CFRFP is ±45°, whereas the ply thickness is 0.2 mm. Araldite 2011 (Huntsman Advanced Material) was used as an adhesive between the nickel and the carbon fiber. Deflection of the master roller mold, which is a very important parameter pertaining to the quality of the R2R process and uniformity of the pressure, was evaluated using Ansys/Explicit non-linear simulation. While metallic assemblies do provide the desired strength, they are too heavy to be suitable in all scenarios. Additionally, deflections are very detrimental to the nanoimprinting process, and deflections in assemblies are desired to be as low as possible. For these reasons, CFRP was chosen as the base material. Because it is a carbon composite material, it has less weight while still having satisfactory strength limits and shows fewer deflections under its own weight. Furthermore, using CFRP also means the assembly is easier to handle. Figure 3 demonstrates the metal sleeve mold and CFRP base roll on a smaller scale.

Our scenario consisted of two rollers under nip loading conditions. The printing roller comprised a hollow carbon composite cylindrical structure on which a thin nickel metal sleeve was joined adhesively using a two-component epoxy-based structural adhesive, Araldite 2011 (Huntsman Advanced Material); the supporting roller consisted of silicon rubber by which the loading condition is applied, along with the rotation. Polymer foil was also passed in between the nip area, and the patterns from the metal were transferred to the foil. Detailed specifications are as follows: the hollow CFRP tube had an outer diameter of 300 mm, a thickness of 27.5 mm, and a length of 1400 mm and was supported by steel supports at both ends, taking the total length of the CFRP roller to 1.9 m. The epoxy adhesive that was applied to the surface of the carbon composite tube had a bond length of 1200 mm and a bond line thickness of 0.1 mm. In the next step, the nickel metal sleeve with a length of 1200 mm and a thickness of 0.25 mm containing the desired patterns to be transferred was adhesively bonded, completing the assembly of the printing roller. However, to complete the nip loading condition, a supporting roller is also required. In this case, it comprised silicon rubber with steel supports at both ends, having an outer diameter of 100 mm. In terms of loading conditions, variable magnitudes of force were applied at both ends of the silicon rubber roller to create the nip pressure for imprinting and assessing the variables. In addition to the application of the force, a rotational velocity of 5 RPM was given to the CFRP roller, which forms frictional contact between the printing and supporting rollers.

### 2.2. Modelling and Simulation

The computer-aided design of the whole assembly was modeled and evaluated in the SOLIDWORKS software, whereas the simulation was conducted on Ansys (Workbench).

The nanoimprinting process, as the name suggests, operates at the nanoscale, at which accuracy and precision are very important. The roll-to-roll process has so far been limited to small scales in terms of the width of the patterns [35]. Researchers have been trying to increase the width of the patterns, but they face the major problem of pressure non-uniformity caused by deflection in the master roller assembly. To resist the bending and reduce the deflections, a lightweight, stiff material with a higher strength-to-weight ratio is required. Therefore, CFRP was selected to be the main part of the base roller due to its desirable attributes, which can perform better than the usual materials, such as steel. Moreover, a number of simulations were performed to assess the viability of the adhesive bond between the CFRP and the nanopatterned nickel mold with loading values ranging from 5 to 50 N. This range of values produces deflections in the mold in the range of nanometers. The adhesive layer was evaluated using the criterion of von Mises stresses, strains, and displacement against an applied force, while the large magnitude of loading values was applied in order to check the adhesive failure limits. Parameters of deflections, applied force and pressure have been carefully selected by keeping in view the system under observation and parameters studied by similar studies [36]. Material properties of the nickel and carbon fiber adherends are listed in Table 1 andTable 2.

#### 2.2.1. Adhesive Material

Different classes of adhesives have been used in structural applications for quite some time in modern industry, owing to their ability to join dissimilar materials with a uniform load distribution, their resistance to corrosion and vibrations, and higher fatigue impedance. For this study, an epoxy adhesive was selected due to its excellent performance in compression. Epoxy adhesives have higher failure limits in compression scenarios rather than tension [37]. This attribute is very desirable in this study. For this study, a two-component epoxy-based structural adhesive, Araldite 2011 (Huntsman Advanced Material), was selected. Its properties are listed in Table 3.

#### 2.2.2. Cohesive Zone Model

The cohesive zone modeling consists of a stress-displacement vital relation of the element, which is utilized in simulating the behavior of the element and the subsequent response on failing. The extent to which the stiffness of the element is degraded is assessed and calculated until it fails. This method of failure analysis is widely practiced for adhesives. Cohesive zone parameters, such as normal critical energy release and shear critical energy release, are listed in Table 1. Similar material properties have been used in numerical simulations throughout the literature [38]. The bilinear constitutive relation of the cohesive element was used in this study to mimic the adhesive layer failure mechanism. The stress displacement behavior of the cohesive element is considered to be linear in the bilinear constitutive relation. This model includes one normal stress component (σN) and two tangential stress components (τs, τt), along with their corresponding displacement components ε (δN, δs, and δt).

(1) $$\left[\begin{array}{c} {\;\sigma }_{N\;}\\ \tau_{s}\\ \tau_{t} \end{array}\right]=\left(K_{N}K_{s}K_{t}\right)\;\;\;\left[\begin{array}{c} {\;\delta }_{N\;\;}\\ \delta_{s}\\ \delta_{t} \end{array}\right]$$

The ε normal and tangential strain components are represented by εN, εs, and εt. The displacement of the element increases with the loading values, and when the displacement reaches the damage value, then the element begins to show signs of failure.

#### 2.2.3. Finite Element Analysis Modelling

To assess the behavior of the cylindrical adhesive lap joint for the current scenario Non-linear Ansys solver was used as materials change stiffness as it deforms. Material non-linearity is included due to the inclusion of materials such as silicon rubber, which is prone to large strain and deformations accompanied by the adhesive layer, while geometric and contact non-linearity effects were also included. The Newton–Raphson method was adopted with an iterative approach along with software using explicit behavior as it is more effective regarding the solution of non-linear analysis [39]. In all finite element analyses, mesh refinement is performed as it provides accurate results. However, this phenomenon is a very computationally demanding and time-consuming process. In order to obtain accurate results, a tradeoff between mesh size and computational time and the cost was made, with the mesh size of the thinnest “adhesive” layer set to 0.01 mm. Important simulation parameters are stated below in Table 4.

#### 2.2.4. Mathematical Modelling

The response of the system to the application of force was modeled mathematically. Different mathematical models, such as linear, exponential, cubic, and higher degree polynomials, were used to model the system, with the magnitude of error serving as the performance criterion for each technique. The input of the system was a range of force values, whereas corresponding deflection values served as the output of the system. After the comparison of the residual’s magnitude, the sixth-degree polynomial produced the smallest residuals. The following equation describes the behavior of the system with optimal accuracy:

(2) $$Y=1.1e^{02}\times z^{6}+1.2e^{2}\times z^{5}+2.6e^{02}\times z^{4}-2.3e^{02}\times z^{3}-1.4e^{02}\times z^{2}+4.2e^{02}\times z+3.8e^{02}$$

where

(3) z=x−2219

**Figure 4:**
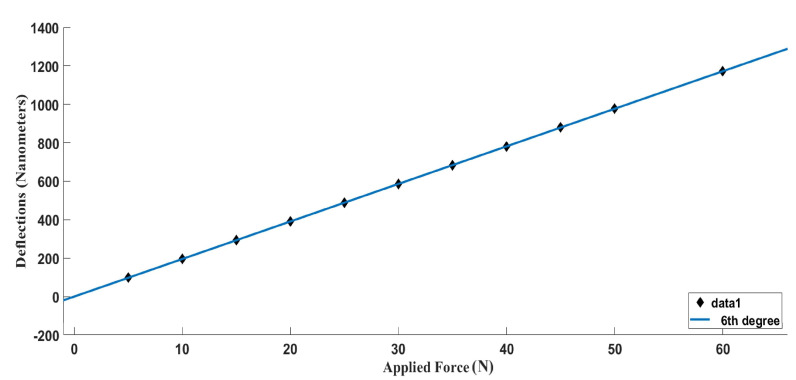
Numerical function plot for deflection values against the incrementally applied force. (Made using MATLAB).

In this expression, ‘Y’ represents the deflections experienced as a result of input force represented by ‘x’. The sixth-degree function gives the value of 5.7 × 10^−3^ residual error, as can be observed in Figure 4.

## 3. Results

**Figure 5:**
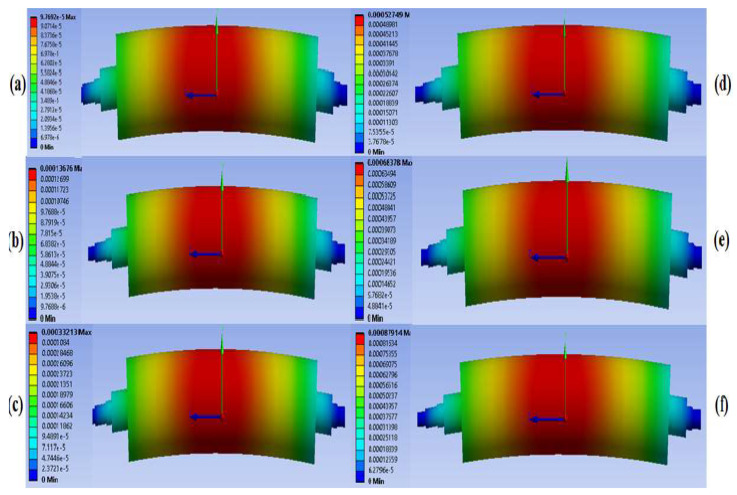
Deflection of master roller assembly (in mm) under different conditions with respect to applied loadings of (**a**) 5 N, (**b**) 10 N, (**c**) 20 N, (**d**) 30 N, (**e**) 40 N, and (**f**) 50 N.

In this study, we carefully measured the deflection of the master roller mold, which is an important parameter influencing the operation’s quality. Deflection is produced in response to the force applied at the ends of the supporting roller during the imprinting process. The magnitude of the deflections in the master roll mold assembly was measured using simulations conducted by varying the applied imprinting force from 5 to 50 N. The minimum value of deflection was found to be 97.69 nm against a loading force of 5 N, as shown in Figure 5a. In the next step, the applied force was increased to 10 N with a corresponding deflection of 136.76 na, as shown in Figure 5b. The magnitude of applied force was increased to 20 N, 30 N, 40 N, and 50 N, as demonstrated in Figure 5c–f, respectively. The respective deflections against the different magnitudes of force are listed in Table 5.

**Figure 6:**
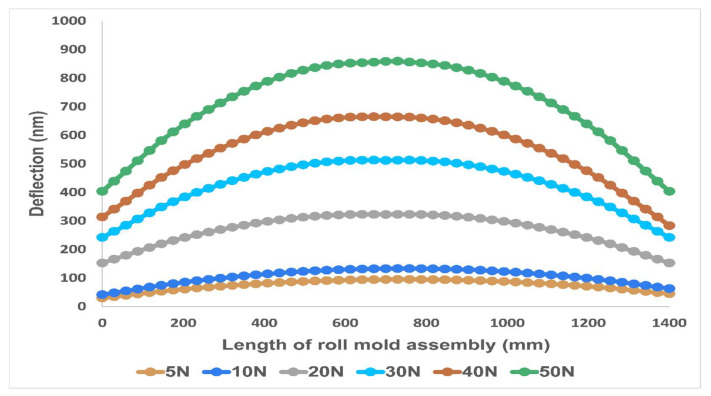
Deflection values (in nanometers) of metallic roll mold assembly along the length.

Figure 6 demonstrates a comparison between measured deflection magnitudes of the master roller assembly of different simulation cases corresponding to the length of the assembly. For cases of 5 N, 10 N, and 20 N, the deflection graph shows a very smooth curve with a low gradient near both edges, with deflection values gradually increasing to a maximum at the center of the metal sleeve. For forces of 30 N, 40 N, and 50 N, the deflection graph shows a steep ascent near both edges which gradually decreases as the maximum value of deflection is reached in the center.

Although the graphs of deflections under different magnitudes of loading show different slopes near both ends, they all followed the same symmetrical trend for the maximum and the minimum values. Table 5 shows the loading values with their corresponding deflection and pressure values.

**Figure 7:**
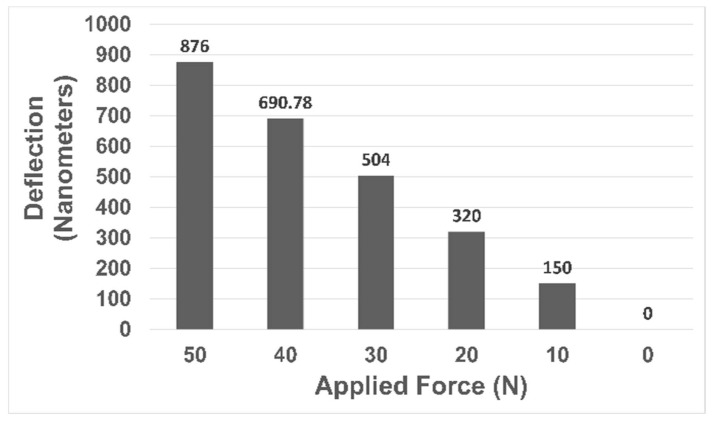
Plot depicting deformation magnitude with respect to applied force.

Nanoimprinting functions at the nanometer scale; therefore, deflections can have an important impact on the properties of the product being manufactured. In Figure 7, the deflections encountered in all cases of FEA analysis are shown plotted against force, where they show a linear trend. Forces of 20 N to 50 N are not practical for use in the nanoimprinting process, as the deflections they cause in the master roller assembly are too large. However, analyses conducted at 10 N show an acceptable range of deflections, up to 136.76 nm. Deflections can be decreased in the 20 to 50 N cases by adopting the strategies suggested in the discussion section.

**Figure 8:**
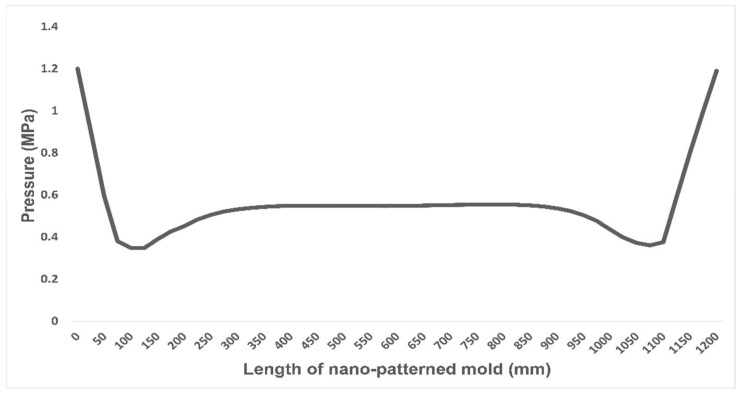
Pressure distribution along the length of the nano mold.

The compressive behavior of the bonded joint depends on the cohesive strength of the adhesive, as well as the adhesion strength between the adhesive and the adherent. As discussed above, the deflection of the master mold assembly during R2R nanoimprinting is very important to the quality of the product being fabricated. For this reason, stress analysis was conducted on the cylindrical adhesive lap joint with loading values that produced acceptable deflection values. In these simulations, variable imprinting loads were applied at the ends of the supporting roller. The magnitude of the stresses that were produced by these loads in the adhesive layer was well below failure stress. Higher loading values were also applied to assess the strength limit of the adhesive bond line. However, in the FEM analysis, it was found that the edges of the adhesive layer in the common area between the master roller and the imprinting roller experienced higher values of stress than the center of the adhesive layer, as shown in Figure 8. This stress behavior is also in agreement with the pressure distribution.

**Figure 9:**
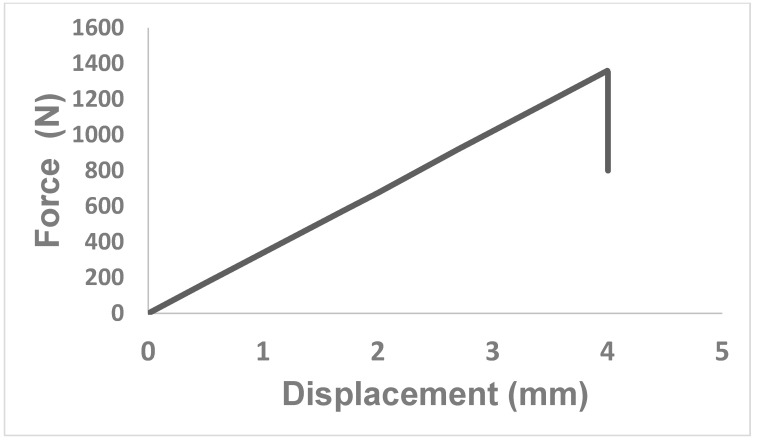
Displacement–force curve for compressive behavior of the adhesive under applied nip force.

We also measured the behavior of the adhesive in response to applied force. The force–displacement relationship and stress–strain response of the adhesive were both found to be linear, as shown in Figure 9. After reaching the failure load of 1350 N with the corresponding displacement of a little above 4 mm, an abrupt drop in the stress and load was observed, indicating the cohesive failure of the adhesive and demonstrating its brittle nature.

In terms of shear stress, the maximum value of shear stress was 1.8 MPa. However, there are always discrepancies present to some degree between the simulation results and experimental results. The reason for this is the abnormalities present in the adhesive layer, such as trapped air bubbles, impurities, dirt, etc. The best way to reduce this difference is to adopt the best available practices in developing the adhesive bond between the adherends by relying on ASTM standards, e.g., D2651-01(2016), D4896-01(2016), D896-04(2017), D6412/D6412M-99(2020), etc.

## 4. Discussion

The fabrication of nano-appliances using a roll-to-roll setup requires minimal deflection, as deflections have a direct influence on the quality of the nano-imprinted products and also decrease the pressure uniformity. The typical height dimensions range from under 100 nm to above 500 nm in practical applications such as anti-reflection films, solar cells, nanofluidic channels, anti-fingerprint films, optical films, and metallic and soft molds [40,41,42,43].

Roll-to-roll nanoimprint lithography has primarily been focused on the production of large and flexible films. However, the development of large cylindrical molds is difficult, as patterns have to be etched onto the surface of the cylindrical metal mold before assembling it onto the base roll. For some specific applications, such as hydrophobic and oleophobic surfaces for marine equipment and large flat displays, the size of the product increases, which in turn increases the roller length [44]. However, increasing the dimensions of the mold also puts the assembly at risk of experiencing more deflection and non-uniformity of pressure due to bending. A continuous large-area R2R nanoimprint system has yet to be shown to work efficiently due to these problems. As can be observed in Figure 7, the deflection of the roller mold assembly increases with increasing force at an undesirable rate. There is a need to decrease the deflection at comparatively higher loading values to ensure good-quality imprinting of patterns along with greater pressure uniformity. Multiple approaches have been suggested for this purpose. One conventional approach is to increase the stiffness of the material, which can be done by using stiffer materials or by increasing the thickness dimensions of the CFRP structure. Another conventional way to minimize deflections is to give little translational room to one side of the support (approximately 1/100th of the total length of the roller) while keeping the other support fixed. The most effective and proven approach is to use an additional supporting roller to minimize the deflections by applying a small magnitude of the force from the opposite side to that of the imprinting force [36].

Our scenario was limited to the application of low magnitude loading values because of the deflection values of the nanopatterned mold. However, it was prudent to push the adhesive bond into a more severe condition to test its strength under higher loading conditions. The deflection was present in the assembly, because of which the edges experienced higher stresses than the middle part of the adhesive layer. This shows that the edges of this layer are vulnerable during nip operations and that failure will occur here first. Moreover, deflection in the assembly also creates stress concentration at the edges of the adhesive layer, which can lead to the failure of the adhesive at comparatively lower load values and, in turn, lower fatigue resistance [45,46]. A decrease in stress concentration will also increase the fatigue limit of the adhesive bond. This can be achieved by increasing the pressure uniformity, which will lead to more uniform stress distribution in the adhesive layer, as discussed in deflection reduction strategies. In many studies, epoxy adhesives have shown similar behavior under compression shear loading scenarios in adhesive joints of different configurations [47,48]. The main reason for the better performance of the adhesive bond under compression is due to the reversal of normal stress from a positive peel in tensile stress cases to the negative direction under compression [47]. An optimum adhesive bond line thickness was selected, as thick bond lines are susceptible to failure at comparatively lower load values owing to defects present in them [49]. It is impossible to completely avoid irregularities in the adhesive bond line. However, increasing the thickness of the adhesive bond increases the probability of flaws occurring that are extremely detrimental to adhesive performance. These faults include voids, air trappings, micro-cracks, etc., and in many studies, thin bond lines have been proven to have better strength limits [50,51].

The main limitations of this study include the computational power of the system on which the FEM simulations were performed. A higher degree of mesh refinement can be achieved with better computational systems, which will result in more accurate results. Moreover, as observed in the pressure profile, it is evident that edges experience higher stress values, which is the cause of stress concentration. This can lead to the failure of the adhesive layer in real scenarios at lower stress levels than predicted in the simulation, as it contains non-linearities. The scope of the current study is limited to simulation-based evaluation mainly due to the high cost of the experimental setup. However, researchers have plans to carry out the actual experiment-based study in the future.

## 5. Conclusions

This study investigated the feasibility of a large-area roll-to-roll nanoimprint setup by observing important factors such as deflections in the master roller assembly and the adhesive bond between the nanopatterned mold and CFRP base roll. In the FEM analysis, deflections found on the imprinting roller as a result of applied nip forces (5–50 N) provide motivation for research to develop procedures on an industrial scale. A number of strategies have been discussed to reduce deflections which have the potential to improve pressure uniformity. In addition, they will also enhance the quality of nano-fabrications, i.e., solar cells, hydrophobic, oleophobic surfaces, etc. Furthermore, adhesive failure analysis was also conducted to evaluate the viability of the bond. The stress levels in the adhesive layer under the applied range of nip forces did not threaten the sustainability of the cylindrical adhesive lap joint. However, the edges displayed comparatively higher stress values. For future studies, the fatigue response of the adhesive bond should be assessed to study its behavior and endurance limit under cyclic loadings.

## Figures and Tables

**Table 1:** Nickel properties.

Property	Density (kg/m^3^)	Young’s Modulus (GPa)	Poisson’s Ratio	Bulk Modulus (GPa)	Shear Modulus (GPa)	Tensile Yield Strength (MPa)
Values	8900	199.5	0.312	176.8	76	150

**Table 2:** Epoxy Carbon UD (230 GPa) Prepreg properties.

Property	Density (kg/m^3^)	Young’s Modulus (GPa)	Poisson’s Ratio	Shear Strength (MPa)	Shear Modulus (GPa)	Tensile Strength (MPa)
Values	1490	121	0.4	60	4.7	2231

**Table 3:** Adhesive properties.

Parameters	Values
Tensile strength	26.36 MPa
Shear traction	23.12 MPa
Elastic modulus	1.802 GPa
Poisson ratio	0.29
GIC (N/mm)	0.25
GIIc and GIIIc (N/mm)	2.61
Viscosity (cP)	40,000

**Table 4:** Simulation parameters.

Parameters	Description
Physics Type	Structural
Physics Preference	Explicit
Solver Type	Iterative
Elements	194,757
Mesh Quality	Fine
Mesh Method	Multizone
Mesh Type	Hexa/prism

**Table 5:** Relationship of deflection and pressure with respect to the applied force.

Force (N)	Deflection (nm)	Pressure (MPa)
50	879.14	0.744
40	683.78	0.59
30	527.49	0.446
20	332.13	0.297
10	136.76	0.1488
5	97.69	0.0844

## Data Availability

In this work, the commercial numerical modeling software ANSYS was used to perform the FEM-based deflection analysis. Interested readers can reproduce the results using the same conditions as listed in the manuscript in the designated software.

## References

[1] Pantelakis S., Tserpes K.I. (2014). Adhesive bonding of composite aircraft structures: Challenges and recent developments. Sci. China Phys. Mech. Astron..

[2] Yacobi B.G., Martin S., Davis K., Hudson A., Hubert M. (2002). Adhesive bonding in microelectronics and photonics. J. Appl. Phys..

[3] Cavezza F., Boehm M., Terryn H., Hauffman T. (2020). A review on adhesively bonded aluminium joints in the automotive industry. Metals.

[4] James B.D., Kimmins K.M., Nguyen M.T., Lausch A.J., Sone E.D. (2021). Attachment of zebra and quagga mussel adhesive plaques to diverse substrates. Sci. Rep..

[5] Heitzer M., Brockhaus J., Kniha K., Merkord F., Peters F., Hölzle F., Goloborodko E., Modabber A. (2021). Mechanical strength and hydrostatic testing of VIVO adhesive in sutureless microsurgical anastomoses: An ex vivo study. Sci. Rep..

[6] Pethrick R.A. (2012). Composite to metal bonding in aerospace and other applications. Welding and Joining of Aerospace Materials.

[7] Borges C.S.P., Marques E.A.S., Carbas R.J.C., Ueffing C., Weißgraeber P., Silva L.D. (2021). Review on the effect of moisture and contamination on the interfacial properties of adhesive joints. Proc. Inst. Mech. Eng. Part C J. Mech. Eng. Sci..

[8] Cruz-G C.E., Akhavan-Safar A., Da Silva L.F.M., Ayatollahi M.R. (2020). On the evaluation of a critical distance approach for failure load prediction of adhesively bonded dissimilar materials. Contin. Mech. Thermodyn..

[9] Barbosa N.G.C., Campilho R.D.S.G., Silva F.J.G., Moreira R.D.F. (2018). Comparison of different adhesively-bonded joint types for mechanical structures. Appl. Adhes. Sci..

[10] Liu W., Xu P., Guo Y., Lin Y., Yin X., Tang G., He M. (2016). *In Situ* growth of highly adhesive surface layer on titanium foil as durable counter electrodes for efficient dye-sensitized solar cells. Sci. Rep..

[11] Murakami S., Sekiguchi Y., Sato C., Yokoi E., Furusawa T. (2016). Strength of cylindrical butt joints bonded with epoxy adhesives under combined static or high-rate loading. Int. J. Adhes. Adhes..

[12] Ozenc M., Sekercioglu T. (2008). Experimental and computational investigation of adhesive joints subjected to impact loads. Kov. Mater..

[13] Nemes O., Lachaud F. (2009). Modeling of cylindrical adhesively bonded joints. J. Adhes. Sci. Technol..

[14] Cognard J.Y., Sohier L., Créac’Hcadec R., Lavelle F., Lidon N. (2012). Influence of the geometry of coaxial adhesive joints on the transmitted load under tensile and compression loads. Int. J. Adhes. Adhes..

[15] Xu W., Li G. (2010). Finite difference three-dimensional solution of stresses in adhesively bonded composite tubular joint subjected to torsion. Int. J. Adhes. Adhes..

[16] Kim Y., Chun Y., Cheon S.S. (2015). Shear directional impact characteristics of adhesively bonded tubular joints. Adv. Compos. Mater..

[17] Parashar A., Mertiny P. (2012). Adhesively bonded composite tubular joints. Int. J. Adhes. Adhes..

[18] Hamoodi-Tabar M., Reza A. (2021). Long-term shear stress distribution in adhesively bonded tubular joints under tensile load using the time-temperature superposition principle. J. Adhes..

[19] Hou W., Xu X., Sang L., Tong L. (2020). Failure of single hat-shaped thin-walled tubular composite T-joints under impact loading. Thin-Walled Struct..

[20] Mustapha F., Sim N.W., Shahrjerdi A. (2012). Investigation of axial crushing behaviour of a composite fuselage model using the cohesive elements. J. Theor. Appl. Mech..

[21] Bharti K., Kumaraswamidhas L.A., Das R.R. (2020). A novel optimization approach for bonded tubular gap K-joints made of FRP composites. Structures.

[22] Baishya N., Das R.R., Panigrahi S.K. (2017). Failure analysis of adhesively bonded tubular joints of laminated FRP composites subjected to combined internal pressure and torsional loading. J. Adhes. Sci. Technol..

[23] Bharti K., Kumaraswamidhas L.A., Das R.R. (2020). Detailed investigation of adhesive fillet tubular T-joint of laminated FRP composite tube under axial compressive load. Weld. World.

[24] Chou S.Y., Krauss P.R., Renstrom P.J. (1995). Imprint of sub-25 nm vias and trenches in polymers. Appl. Phys. Lett..

[25] Atthi N., Dielen M., Sripumkhai W., Pattamang P., Meananeatra R., Saengdee P., Thongsook O., Ranron N., Pankong K., Uahchinkul W. (2021). Fabrication of High Aspect Ratio Micro-Structures with Superhydrophobic and Oleophobic Properties by Using Large-Area Roll-to-Plate Nanoimprint Lithography. Nanomaterials.

[26] Li Y., John J., Kolewe K.W., Schiffman J.D., Carter K.R. (2015). Scaling up nature: Large area flexible biomimetic surfaces. ACS Appl. Mater. Interfaces.

[27] Shao J., Chen X., Li X., Tian H., Wang C., Lu B. (2019). Nanoimprint lithography for the manufacturing of flexible electronics. Sci. China Technol. Sci..

[28] Badiger P.V., Desai V., Ramesh M.R., Prajwala B.K., Raveendra K. (2018). Effect of cutting parameters on tool wear, cutting force and surface roughness in machining of MDN431 alloy using Al and Fe coated tools. Mater. Res. Express.

[29] Badiger P.V., Desai V., Ramesh M., Joladarashi S., Gourkar H. (2018). Tribological behaviour of monolayer and multilayer Ti-based thin solid films deposited on alloy steel. Mater. Res. Express.

[30] Badiger P.V., Desai V., Ramesh M., Prajwala B., Raveendra K. (2019). Cutting forces, surface roughness and tool wear quality assessment using ANN and PSO approach during machining of MDN431 with TiN/AlN-coated cutting tool. Arab. J. Sci. Eng..

[31] Badiger P.V., Desai V., Ramesh M. (2017). Development and characterization of Ti/TiC/TiN coatings by cathodic arc evaporation technique. Trans. Indian Inst. Met..

[32] Samneang H., Kumar L., Zafar A., Ali M.U., Zahid T., Bibi S., Ahmad M.S., Ghafoor U., Selvaraj J. (2021). A systematic indoor and outdoor study of the effect of particle size and concentration of TiO_2_ in improving solar absorption for solar still application. Front. Mater..

[33] Farooq U., Ali M.U., Hussain S.J., Ahmad M.S., Zafar A., Ghafoor U., Subhani T. (2021). Improved Ablative Properties of Nanodiamond-Reinforced Carbon Fiber–Epoxy Matrix Composites. Polymers.

[34] Ahmad M.S., Han S.S., Zafar A., Ghafoor U., Rahim N.A., Ali M.U., Rim Y.S. (2021). Indoor and outdoor performance study of metallic zinc particles in black paint to improve solar absorption for solar still application. Coatings.

[35] Lim H., Choi K.B., Kim G., Lee S., Park H., Ryu J., Jung S., Lee J. (2014). Roll-to-roll nanoimprint lithography for patterning on a large-area substrate roll. Microelectron. Eng..

[36] Kim G.E., Kim H., Woo K., Kang Y., Lee S.-H., Jeon Y., Lee M.G., Kwon S. (2021). Uniform Pressing Mechanism in Large-Area Roll-to-Roll Nanoimprint Lithography Process. Appl. Sci..

[37] Choudhury M.R., Debnath K. (2019). Experimental analysis of tensile and compressive failure load in single-lap bolted joint of green composites. Compos. Struct..

[38] Paygozar B., Dizaji S., da Silva L.F.M. (2020). Technology. Bonding dissimilar materials via adhesively bonded spot-welded joints: Cohesive zone model technique. J. Adhes. Sci. Technol..

[39] Mustapha F., Shahrjerdi A., Sim N.W. (2012). Engineering. Finite element validation on adhesive joint for composite fuselage model. J. Braz. Soc. Mech. Sci. Eng..

[40] Lee J., Park H.-H., Choi K.-B., Kim G., Lim H. (2014). Fabrication of hybrid structures using UV roll-typed liquid transfer imprint lithography for large areas. Microelectron. Eng..

[41] Liu C.-H., Sung C.-K., Chang E.-C., Lo C.-Y., Fu C.-C. (2013). Fabricating a silver soft mold on a flexible substrate for roll-to-roll nanoimprinting. IEEE Trans. Nanotechnol..

[42] Rank A., Lang V., Lasagni A.F. (2017). High-speed roll-to-roll hot embossing of micrometer and sub micrometer structures using seamless direct laser interference patterning treated sleeves. Adv. Eng. Mater..

[43] Zhang C., Yi P., Peng L., Lai X., Ni J. (2015). Fabrication of moth-eye nanostructure arrays using roll-to-roll UV-nanoimprint lithography with an anodic aluminum oxide mold. IEEE Trans. Nanotechnol..

[44] Ahn S.H., Guo L.J. (2009). Large-area roll-to-roll and roll-to-plate nanoimprint lithography: A step toward high-throughput application of continuous nanoimprinting. ACS Nano.

[45] Mathias J.-D., Lemaire M.J. (2013). Reliability analysis of bonded joints with variations in adhesive thickness. J. Adhes. Sci. Technol..

[46] Sancaktar E. (1995). Fracture aspects of adhesive joints: Material, fatigue, interphase, and stress concentration considerations. J. Adhes. Sci. Technol..

[47] Choudhury M.R., Debnath K. (2020). Experimental analysis of tensile and compressive failure load in single-lap adhesive joint of green composites. Int. J. Adhes. Adhes..

[48] Challita G., Othman R., Khalil K. (2016). Compression and shear behavior of epoxy SA 80 bulk adhesive over wide ranges of strain rate. J. Polym. Eng..

[49] Zhang D., Huang Y. (2021). Influence of surface roughness and bondline thickness on the bonding performance of epoxy adhesive joints on mild steel substrates. Prog. Org. Coat..

[50] Seo D.W., Yoon H.C., Jeon Y.B., Kim H.J., Lim J.K. (2004). Effect of overlap length and adhesive thickness on stress distribution in adhesive bonded single-lap joints. Key Engineering Materials.

[51] Arenas J.M., Narbón J.J., Alía C.J. (2010). Optimum adhesive thickness in structural adhesives joints using statistical techniques based on Weibull distribution. Int. J. Adhes..

